# Association of Pharmacological Treatments and Hospitalization and Death in Individuals With Amphetamine Use Disorders in a Swedish Nationwide Cohort of 13 965 Patients

**DOI:** 10.1001/jamapsychiatry.2022.3788

**Published:** 2022-11-16

**Authors:** Milja Hartikainen, Heidi Taipale, Antti Tanskanen, Ellenor Mittendorfer-Rutz, Markku Lähteenvuo, Jari Tiihonen

**Affiliations:** 1 Department of Forensic Psychiatry, University of Eastern Finland, Niuvanniemi Hospital, Kuopio, Finland; 2 Division of Insurance Medicine, Department of Clinical Neuroscience, Karolinska Institutet, Stockholm, Sweden; 3 School of Pharmacy, University of Eastern Finland, Kuopio, Finland; 4 Department of Clinical Neuroscience, Karolinska Institutet & Centre for Psychiatry Research, Stockholm Health Care Services, Region Stockholm, Stockholm, Sweden; 5 Neuroscience Center, University of Helsinki, Helsinki, Finland

## Abstract

**Importance:**

There are no medications approved by authorities for the treatment of amphetamine or methamphetamine dependence, and studies investigating the effectiveness of pharmacological treatments in hard outcomes, such as hospitalization and death, are lacking.

**Objective:**

To investigate the association between pharmacotherapies and hospitalization and mortality outcomes in persons with amphetamine or methamphetamine use disorder.

**Design, Setting, and Participants:**

This nationwide register-based cohort study was conducted from July 2006 to December 2018 with a median (IQR) follow-up time of 3.9 (1.0-6.1) years. Data were analyzed from December 1, 2021, to May 24, 2022. All residents aged 16 to 64 years living in Sweden with a registered first-time diagnosis of amphetamine or methamphetamine use disorder and without previous diagnoses of schizophrenia or bipolar disorder were identified from nationwide registers of inpatient care, specialized outpatient care, sickness absence, and disability pension.

**Exposures:**

Medications for substance use disorders (SUDs) or for attention-deficit/hyperactive disorder, mood stabilizers, antidepressants, benzodiazepines and related drugs, and antipsychotics. Medication use vs nonuse was modeled with the PRE2DUP (from prescription drug purchases to drug use periods) method.

**Main Outcomes and Measures:**

Primary outcomes were hospitalization due to SUD and any hospitalization or death, which were analyzed using within-individual models by comparing use and nonuse periods of 17 specific medications or medication classes in the same individual to minimize selection bias. The secondary outcome was all-cause mortality, studied using between-individual analysis as traditional Cox models.

**Results:**

There were 13 965 individuals in the cohort (9671 [69.3%] male; mean [SD] age, 34.4 [13.0] years). During follow-up, 7543 individuals (54.0%) were taking antidepressants, 6101 (43.7%) benzodiazepines, 5067 (36.3%) antipsychotics, 3941 (28.2%) ADHD medications (1511 [10.8%] were taking lisdexamphetamine), 2856 (20.5%) SUD medications, and 1706 (12.2%) mood stabilizers. A total of 10 341 patients (74.0%) were hospitalized due to SUDs, 11 492 patients (82.3%) were hospitalized due to any cause or died, and 1321 patients (9.5%) died of any cause. Lisdexamphetamine was the only medication in this study that was significantly associated with a decrease in risk of 3 outcomes (adjusted hazard ratio [aHR], 0.82; 95% CI, 0.72-0.94 for SUD hospitalization; aHR, 0.86; 95% CI, 0.78-0.95 for any hospitalization or death; aHR, 0.43; 95% CI, 0.24-0.77 for all-cause mortality). Methylphenidate use also was associated with lower all-cause mortality (aHR, 0.56; 95% CI, 0.43-0.74). Use of benzodiazepines was associated with a significantly higher risk of SUD hospitalization (aHR, 1.17; 95% CI, 1.12-1.22), any hospitalization or death (aHR, 1.20; 95% CI, 1.17-1.24), and all-cause mortality (aHR, 1.39; 95% CI, 1.20-1.60). Use of antidepressants or antipsychotics was associated with a slight increase in risk of SUD hospitalization (aHR, 1.07; 95% CI, 1.03-1.11 and aHR, 1.05; 95% CI, 1.01-1.09) as well as any hospitalization or death (aHR, 1.10; 95% CI, 1.06-1.14 and aHR, 1.06; 95% CI, 1.03-1.10, respectively).

**Conclusions and Relevance:**

In this study, use of lisdexamphetamine was associated with improved outcomes in persons with amphetamine or methamphetamine use disorders, encouraging the conduct of randomized clinical trials. Prescription benzodiazepine use was associated with poor outcomes.

## Key Points

**Question:**

What is the association between pharmacological treatments and hospitalization and mortality outcomes in individuals with amphetamine use disorders?

**Findings:**

In this Swedish nationwide cohort study of 13 965 individuals, lisdexamphetamine was significantly associated with a decrease in risk of hospitalization due to substance use disorder, any hospitalization or death, and all-cause mortality.

**Meaning:**

In this study, lisdexamphetamine was consistently associated with improved outcomes in individuals with amphetamine use disorders, while other pharmacological treatments were not, encouraging the conduct of randomized clinical trials.

## Teaser

This cohort study evaluates the association of commonly used pharmacological treatments with hospitalization and mortality outcomes in individuals with amphetamine use disorders.

## Introduction

Amphetamines are the second most used illicit drugs worldwide and amphetamine-related hospitalizations are increasing substantially.^1,2^ There is an elevated risk of infections and mental disorders associated with methamphetamine or amphetamine use disorders (MAUD).^1,3^ People with MAUD are also at higher risk of mortality compared with the general population, mainly from directly drug-related deaths, but also due to suicide, homicide, cardiovascular disease, and injuries.^4,5^ Amphetamine use is associated with aggressive behavior and criminality, which also indirectly lead to morbidity and mortality.^6^ Mortality related to amphetamine or methamphetamine use is increasing^7,8^ and has doubled over the past decade, possibly indicating the next substance use crisis.^9^ According to the European Monitoring Centre for Drugs and Drug Addiction Sweden Country Drug Report 2019,^10^ amphetamines were the third most commonly used illicit drugs, and 1.2% of young adults aged 17 to 34 years were taking them. Concerning all the harm and costs that MAUD cause for the individual and society, effective treatments seem essential.^11^ However, there are currently no approved pharmacological interventions available for treating MAUD.^6^ Recent meta-analyses have investigated the effectiveness of antidepressants, antipsychotics, psychostimulants, anticonvulsants, and opioid agonists and antagonists^3,6^ and suggest that there are some promising candidates for the treatment of MAUD, yet convincing evidence is lacking.^3^ Treatment with the combination of extended-release injectable naltrexone and daily oral extended-release bupropion resulted in a low, but higher than placebo, response for methamphetamine-negative urine samples.^12^ In addition, the antidepressant mirtazapine has been reported to reduce methamphetamine use when combined with substance use counseling.^13^ The most consistent positive findings have been demonstrated with stimulant agonists (dexamphetamine^14,15^ and methylphenidate^16,17,18^), naltrexone,^19,20^ and topiramate,^21^ whereas antidepressants have shown less consistent results in reducing amphetamine use.^3^ A recent systematic review and meta-analysis^22^ evaluated agonist-based pharmacological interventions (similarly as used in opioid and tobacco use disorders) and found that prescription psychostimulants had a beneficial effect to promote abstinence in persons with stimulant use disorders. Dexamphetamine has similar neurochemical and behavioral effects to methamphetamine,^23^ and it has been used as an off-label treatment for MAUD. Lisdexamphetamine is a pharmacologically inactive prodrug of dexamphetamine. It presents a candidate pharmacotherapy for MAUD and seems relatively safe and well tolerated.^24^ However, studies tend to be limited by small sample sizes in defined populations and by low treatment retention or completion rates.^3^

To our knowledge, no studies have investigated the effectiveness of pharmacological treatments concerning hard outcomes, such as hospitalization and death. We aimed to investigate the association of various pharmacotherapies in persons with MAUD with hospitalization due to substance use disorder (SUD) and any hospitalization or death as main outcomes and mortality due to all causes as the secondary outcome.

## Methods

Nationwide register-based data were used to conduct a population-based cohort study of patients with MAUD. The project was approved by the Regional Ethics Board of Stockholm (decision 2007/762-31). No informed consent is required for register-based studies using anonymized data.

### Study Population

Data were gathered prospectively from nationwide Swedish registers, including the National Patient Register, the Causes of Death Register, the Longitudinal Integration Database for Health Insurance and Labor Market Studies register, and the Micro Data for Analyses of Social Insurance (MiDAS) register. Drug use data were gathered from the Prescribed Drug Register (PDR) from July 2005 to December 2018. The data analysis was conducted from December 1, 2021, to May 24, 2022.

All residents aged 16 to 64 years living in Sweden with a registered first-time treatment contact due to MAUD (*International Statistical Classification of Diseases and Related Health Problems, Tenth Revision* [*ICD-10*] codes F15.0-15.9, other stimulant use, including amphetamine and methamphetamine) between July 1, 2006, and December 31, 2018, were included in this study. They were identified from inpatient, specialized outpatient, sickness absence, and disability pension (MiDAS) registers. Individuals were chosen based on not having a previous diagnosis of schizophrenia or bipolar disorder. All Swedish residents have been assigned a unique personal identification number, which enabled linkage between various registers.

### Exposures

Medication use information in the PDR is categorized according to the Anatomical Therapeutic Chemical classification.^25^ Drugs were categorized as medications for SUDs, medications for attention-deficit/hyperactive disorder (ADHD), mood stabilizers, antidepressants, benzodiazepines and related drugs, and antipsychotics (eMethods in the Supplement). Each medication class was compared with nonuse of that class unless otherwise stated. Medication use periods (ie, when medication use started and ended) were constructed using the PRE2DUP (from prescription drug purchases to drug use periods) method^26^ (eMethods in the Supplement).

### Outcomes

The main outcome measures were hospitalization due to SUD (*ICD-10* codes F10-F19 as a main diagnosis) and hospitalization due to any cause or death. The secondary outcome was all-cause mortality.

### Covariates

Within-individual analyses were adjusted for temporal order of treatments and time since cohort entry (eTable 1 in the Supplement). Between-individual analyses were additionally adjusted for baseline covariates age, sex, education, granted disability pension, long-term sickness absence during previous year (more than 90 days), and time-varying covariates, including medication-related comorbidities (eTable 1 in the Supplement).

### Statistical Analysis

Main outcomes were treated as recurrent events and analyzed with the within-individual Cox regression model^27,28^ (eMethods in the Supplement). A within-individual model was also used in sensitivity analysis on lisdexamphetamine dose categories^29^ (as time-varying dose, measured in defined daily dose [DDD]) (eMethods in the Supplement) and in the analysis, where the first 30 days after medication use started were omitted (omission analysis). The within-individual model is a stratified Cox regression model in which each individual formed his or her own stratum, which reduces selection bias. All-cause mortality was analyzed with traditional multivariate-adjusted Cox regression model as between-individual analysis (eMethods in the Supplement). Follow-up started at the first diagnosis of MAUD and ended at death, emigration, diagnosis of schizophrenia or bipolar disorder, or end of study follow-up (December 31, 2018). Statistical significance was set at .05 using Benjamini-Hochberg false discovery rate method on a per graph basis. The results are reported as adjusted hazard ratios (aHRs) with 95% CIs.

## Results

### Cohort Characteristics

In the total cohort, including 13 965 persons with a diagnosis of MAUD, 9671 individuals (69.3%) were men, and the mean (SD) age was 34.4 (13.0) years. The median (IQR) follow-up time was 3.9 (1.0-6.1) years. During follow-up, 7543 individuals (54.0%) were taking antidepressants, 6101 (43.7%) benzodiazepines, 5067 (36.3%) antipsychotics, 3941 (28.2%) ADHD medications (1511 [10.8%] were taking lisdexamphetamine) 2856 (20.5%) SUD medications, and 1706 (12.2%) mood stabilizers. The number of individuals taking each studied drug are shown in eTable 2 in the Supplement. A total of 4059 patients (29.1%) had work income during the calendar year before cohort entry, 3292 (23.6%) were unemployed for 1 to 180 days, 890 (6.4%) for more than 180 days, 889 (6.4%) for more than 90 days sickness absence, and 2082 (14.9%) were receiving a disability pension at cohort entry. Overall, 4075 participants (29.2%) were diagnosed with alcohol use disorder, 1791 (12.8%) with sedative use disorder, 1623 (11.6%) with opioid use disorder, and 4728 (33.9%) with other psychoactive multiuse disorder. Altogether, 2690 (19.3%) had anxiety disorder, 1843 (13.2%) depression, and 1657 (11.9%) ADHD at baseline. At the end of follow-up, 3160 individuals (22.6%) were diagnosed with ADHD.

### Outcomes

#### Risk of SUD Hospitalization

**Figure 1.**
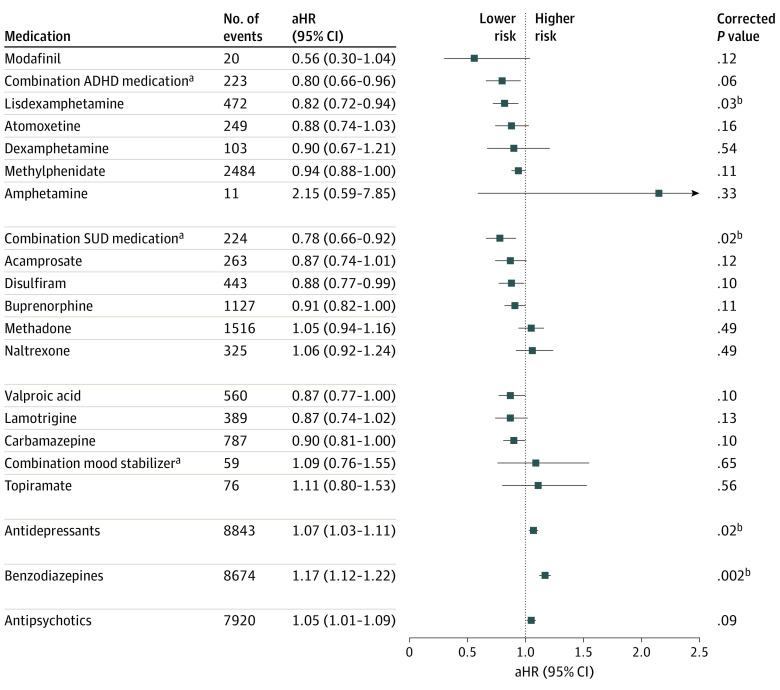
Adjusted Hazard Ratios (aHRs) and 95% CIs for the Risk of Hospitalization Due to Substance Use Disorder (SUD) During Pharmacotherapy Compared With Nonuse of the Medication Class in Within-Individual Analyses ADHD indicates attention-deficit/hyperactive disorder. ^a^Refers to the concomitant use of 2 or more medications. ^b^Results significant after Benjamini-Hochberg false discovery rate correction for multiple comparisons at a .05 threshold.

During follow-up, 10 341 patients (74.0%) were hospitalized due to SUDs. The use of lisdexamphetamine (aHR, 0.82; 95% CI, 0.72-0.94, compared with ADHD medication nonuse), as well as polytherapy of SUD medications (aHR, 0.78; 95% CI, 0.66-0.92, compared with nonuse of SUD medications) were associated with significantly lower risk of SUD hospitalization in within-individual analysis (Figure 1). The results were similar in the 30-day omission analysis and, in addition to lisdexamphetamine, the use of valproic acid was associated with a 13% lower risk of SUD hospitalization (eTable 3 in the Supplement). In between-individual analyses, the use of lisdexamphetamine (aHR, 0.75; 95% CI, 0.66-0.85), combination of ADHD medications (aHR, 0.82; 95% CI, 0.70-0.95), and methylphenidate (HR, 0.90; 95% CI, 0.86-0.95) were associated with reduced risk of SUD hospitalization compared with nonuse of ADHD medications (Table 1). The use of antidepressants (aHR, 1.07; 95% CI, 1.03-1.11) and benzodiazepines (aHR, 1.17; 95% CI, 1.12-1.22) were associated with a significantly increase in risk of SUD hospitalization (Figure 1) and the results remained similar in the omission-analysis (eTable 3 in the Supplement) and in the between-individual analysis (Table 1). In between-individual analysis, also the use of methadone (aHR, 1.25; 95% CI, 1.15-1.36) and antipsychotics (aHR, 1.19; 95% CI, 1.15-1.23) were associated with an increase in risk of SUD hospitalization, and the result was similar for antipsychotics in the omission analysis. Of specific antidepressants, the use of mirtazapine (aHR, 1.08; 95% CI, 1.00-1.15), venlafaxine (aHR, 1.13; 95% CI, 1.02-1.25), and citalopram (HR, 1.14; 95% CI, 1.00-1.29) were associated with an increase in risk of SUD hospitalization, and none of the most used antidepressants were associated with reduced risk (eTable 4 in the Supplement).

**Table 1.** Adjusted Risk of Hospitalization Due to Substance Use Disorder (SUD) and Any Hospitalization or Death in Traditional Between-Individual Cox Model Associated With Use of Medication vs Nonuse of Medication Class

Medication	SUD hospitalization			Any hospitalization or death		
	Events, No.	aHR^a^ (95% CI)	Nominal *P* value	Events, No.	aHR^a^ (95% CI)	Nominal *P* value
SUD medications						
Disulfiram	443	0.90 (0.80-1.01)	.08	649	0.95 (0.86-1.06)	.36
Acamprosate	263	1.00 (0.85-1.17)	.99	344	0.96 (0.84-1.10)	.55
Naltrexone	325	1.14 (0.98-1.33)	.10	406	1.07 (0.92-1.25)	.38
Buprenorphine	1127	1.02 (0.93-1.11)	.75	1332	0.98 (0.90-1.06)	.57
Methadone	1516	1.25 (1.15-1.36)	<.001^b^	1807	1.28 (1.18-1.40)	<.001^b^
≥2 SUD medications	224	0.89 (0.77-1.04)	.13	270	0.91 (0.79-1.05)	.19
Mood stabilizers						
Carbamazepine	787	1.11 (1.01-1.22)	.03	1140	1.14 (1.05-1.23)	.001^b^
Valproic acid	560	0.96 (0.85-1.07)	.44	954	1.08 (0.99-1.18)	.09
Lamotrigine	389	0.92 (0.82-1.03)	.14	787	1.11 (1.00-1.24)	.05
Topiramate	76	0.97 (0.78-1.21)	.81	161	1.14 (0.89-1.46)	.31
≥2 Mood stabilizers	59	1.16 (0.87-1.57)	.32	110	1.18 (0.94-1.50)	.16
ADHD medication						
Amphetamine	11	0.72 (0.44-1.17)	.18	26	0.91 (0.64-1.30)	.61
Dexamphetamine	103	0.83 (0.57-1.21)	.33	222	0.88 (0.72-1.08)	.23
Methylphenidate	2484	0.90 (0.86-0.95)	<.001^b^	4198	0.94 (0.90-0.99)	.01^b^
Modafinil	20	0.72 (0.52-0.99)	.046	49	0.92 (0.72-1.18)	.51
Atomoxetine	249	0.90 (0.78-1.04)	.15	372	0.90 (0.80-1.01)	.06
Lisdexamphetamine	472	0.75 (0.66-0.85)	<.001^b^	909	0.86 (0.78-0.94)	<.001^b^
≥2 ADHD medications	223	0.82 (0.70-0.95)	.007^b^	428	0.89 (0.81-0.99)	.04
Benzodiazepines	8674	1.15 (1.11-1.19)	<.001^b^	15 118	1.23 (1.19-1.26)	<.001^b^
Antipsychotics	7920	1.19 (1.15-1.23)	<.001^b^	11 977	1.23 (1.20-1.27)	<.001^b^
Antidepressants	8843	1.06 (1.02-1.10)	<.001^b^	14 551	1.10 (1.07-1.13)	<.001^b^

Abbreviations: ADHD, attention-deficit/hyperactive disorder; aHR, adjusted hazard ratio.

^a^ Adjusted for other medication use (opioid and nonopioid analgesics, cardiovascular medications, alimentary tract and metabolism medications, and antiepileptic drugs), number of previous hospitalizations due to methamphetamine use disorders, comorbidities (cardiovascular disease, diabetes, asthma or chronic obstructive pulmonary disease, previous cancer, kidney disease, previous suicide attempt, SUD other than methamphetamine use disorders, depression, anxiety disorder, ADHD), and sociodemographic factors (age, sex, and education) with nonuse of medications as a reference.

^b^ Results significant after Benjamini-Hochberg false discovery rate correction for multiple comparisons at a .05 threshold.

#### Risk of Any Hospitalization or Death

**Figure 2.**
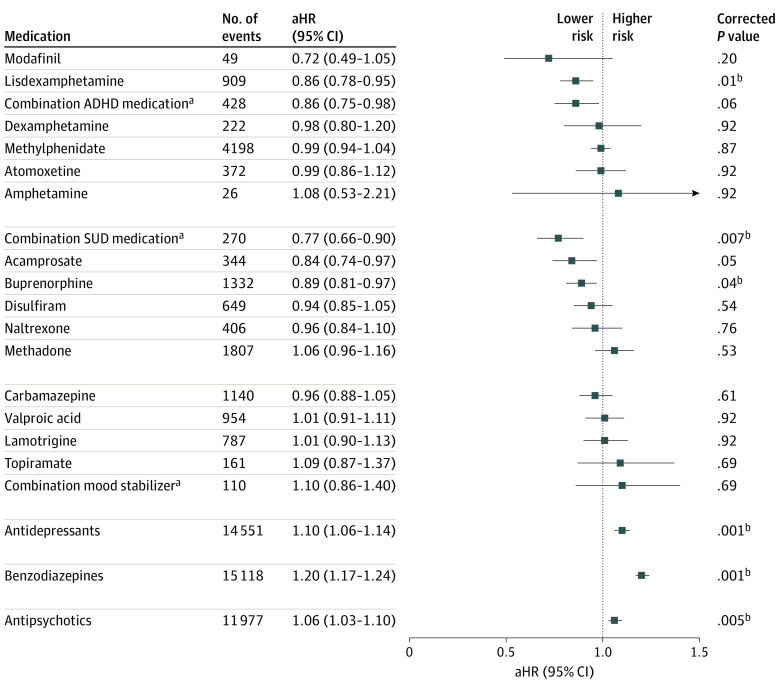
Risk of Hospitalization Due to Any Cause or Death During Use of Pharmacotherapy Compared With Nonuse of the Medication Class in Within-Individual Analyses ADHD indicates attention-deficit/hyperactive disorder; aHR, adjusted hazard ratio; SUD, substance use disorder. ^a^Refers to the concomitant use of 2 or more medications. ^b^Results significant after Benjamini-Hochberg false discovery rate correction for multiple comparisons at a .05 threshold.

During follow-up, 11 492 patients (82.3%) were hospitalized due to any cause or died. The use of a combination of 2 or more SUD medications (aHR, 0.77; 95% CI, 0.66-0.90), lisdexamphetamine (aHR, 0.86; 95% CI, 0.78-0.95), and buprenorphine (aHR, 0.89; 95% CI, 0.81-0.97) were associated with significantly lower risk of any hospitalization or death compared with periods when the same individual was not taking the studied medication class (Figure 2). In the omission analyses, the use of lisdexamphetamine and the combination of 2 or more ADHD medications were associated with a lower risk of any hospitalization or death (eTable 3 in the Supplement). In between-individual analyses, the use of lisdexamphetamine (aHR, 0.86; 95% CI, 0.78-0.94) and methylphenidate (aHR, 0.94; 95% CI, 0.90-0.99) were associated with a lower risk of any hospitalization or death compared with ADHD medication nonuse (Table 1). The use of antidepressants (aHR, 1.10; 95% CI, 1.06-1.14), benzodiazepines (aHR, 1.20; 95% CI, 1.17-1.24), and antipsychotics (aHR, 1.06; 95% CI, 1.03-1.10) were associated with an increase in risk of any hospitalization or death (Figure 2), and the results were similar in the omission analysis (eTable 3 in the Supplement) and in between-individual analysis (Table 1). In between-individual analysis, the use of methadone (aHR, 1.28; 95% CI, 1.18-1.40) and carbamazepine (HR, 1.14; 95% CI, 1.05-1.23) were associated with a significant increase in risk of any hospitalization or death. In the sensitivity analysis for the most used antidepressants, none of the studied antidepressants were associated with favorable outcomes. The use of mirtazapine (aHR, 1.09; 95% CI, 1.02-1.15), venlafaxine (aHR, 1.17; 95% CI, 1.07-1.26), citalopram (aHR, 1.15; 95% CI, 1.05-1.27), fluoxetine (aHR, 1.13; 95% CI, 1.02-1.24), and paroxetine (aHR, 1.19; 95% CI, 1.00-1.43) were associated with an increase in risk of death or hospitalization due to any cause, and none of antidepressants was associated with a lower risk (eTable 4 in the Supplement). The results for the specific combinations of ADHD and SUD medications are shown in eTable 5 in the Supplement.

As a sensitivity analysis for the main outcomes, we performed subgroup analyses, where the use of lisdexamphetamine was stratified by dose categories (<45 mg/d, 45-<65 mg/d, 65-<85 mg/d, and ≥85 mg/d). The risk of SUD hospitalization and the risk of any hospitalization or death were lower in the dose categories 45 to less than 65 mg/d (a reduction of 30% and 23%, respectively) and 65 to less than 85 mg/d (a reduction of 25% and 21%, respectively) compared with nonuse of lisdexamphetamine (Table 2).

**Table 2.** Risk of Outcomes Associated With Use of Lisdexamphetamine Compared With Nonuse of Lisdexamphetamine in Within-Individual Model Stratified by Dose Categories in Defined Daily Doses (DDDs)

	DDD/d	Events, No.	Individuals, No.	Person-years	aHR (95% CI)
**Risk of hospitalization due to substance use disorder**					
Lisdexamphetamine by dose categories					
<45 mg/d	<1.50	72	457	185	1.10 (0.80-1.52)
45 to <65 mg/d	1.50 to <2.17	86	425	308	0.70 (0.52-0.93)
65 to <85 mg/d	2.17 to <2.83	117	399	394	0.75 (0.57-0.99)
≥85 mg/d	≥2.83	197	525	546	0.83 (0.67-1.03)
**Risk of hospitalization due to any cause or death**					
Lisdexamphetamine by dose categories					
<45 mg/d	<1.50	124	455	185	1.02 (0.80-1.30)
45 to <65 mg/d	1.50 to <2.17	167	423	308	0.77 (0.62-0.95)
65 to <85 mg/d	2.17 to <2.83	246	398	392	0.79 (0.64-0.96)
≥85 mg/d	≥2.83	372	517	542	0.92 (0.78-1.07)

Abbreviation: aHR, adjusted hazard ratio.

#### Risk of All-Cause Mortality

**Figure 3.**
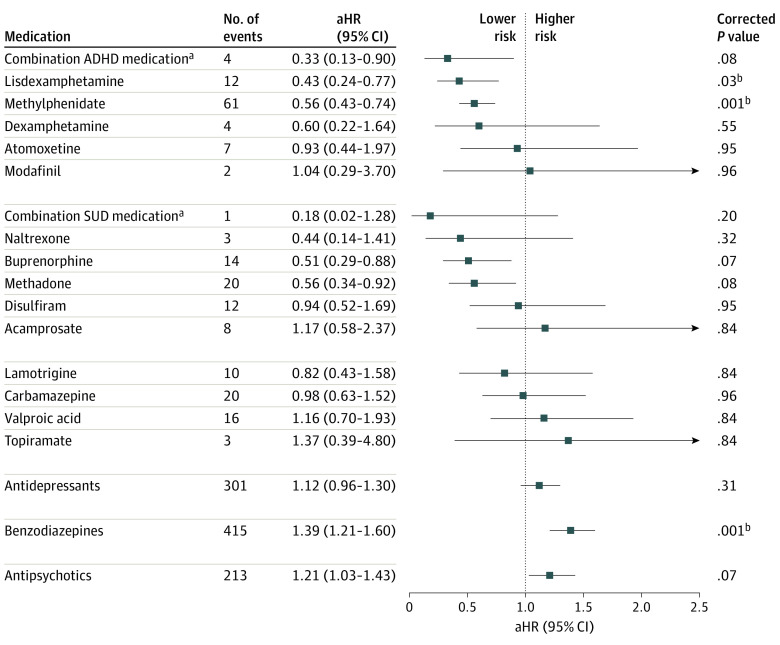
Adjusted Risk of All-Cause Mortality Associated With Medication Use vs Medication Class Nonuse in Between-Individual Analyses (Traditional Cox Model) The use of amphetamine and combination of mood stabilizers were not analyzed due to zero events. ADHD indicates attention-deficit/hyperactive disorder; aHR, adjusted hazard ratio; SUD, substance use disorder. ^a^Refers to the concomitant use of 2 or more medications. ^b^Results significant after Benjamini-Hochberg false discovery rate correction for multiple comparisons at a .05 threshold.

During follow-up, 1321 patients (9.5%) died of any cause. The use of lisdexamphetamine (aHR, 0.43; 95% CI, 0.24-0.77) and methylphenidate (HR, 0.56; 95% CI, 0.43-0.74) were associated with a significantly lower risk of death due to any cause. The use of benzodiazepines (aHR, 1.39; 95% CI, 1.21-1.60) was associated with a significant increase in risk of death (Figure 3). The results were similar in the analysis where the outcome was death due to overdose. In addition to lisdexamphetamine (aHR 0.34, 95% CI, 0.14-0.82) and methylphenidate (HR, 0.60; 95% CI, 0.42-0.85), the use of buprenorphine (aHR, 0.32; 95% CI, 0.14-0.73) and methadone (aHR, 0.44; 95% CI, 0.21-0.93) were also associated with a lower risk of death due to overdose. The use of benzodiazepines (aHR, 1.74; 95% CI, 1.40-2.17) and antipsychotics (aHR, 1.29; 95% CI, 1.02-1.64) were associated with an increase in risk of death due to overdose (eTable 6 in the Supplement).

## Discussion

To the best of our knowledge, no other cohort study has investigated the association of pharmacological treatments and outcomes in patients with MAUD during a long-term follow-up period. This study provides insight concerning the association of different medications, generally used in persons with MAUD, with long-term health outcomes, such as risk of hospitalization and death. We found that, compared with personal nonuse periods, lisdexamphetamine was the only medication studied that was associated with a statistically significant beneficial finding in all 3 outcomes (SUD hospitalization, any hospitalization or death, and all-cause mortality). Benzodiazepines, antidepressants, and antipsychotics were associated with an increase in risk of any hospitalization or death. Benzodiazepines and antidepressants were also associated with an increase in risk of SUD hospitalization and the use of benzodiazepines was associated with a higher risk of death.

Currently there are no officially approved pharmacotherapies for MAUD and, despite promising medication candidates, studies are often limited by small and selected cohorts as well as low treatment retention or completion rates. The most consistent positive findings have been demonstrated with stimulant-agonist treatments as well as naltrexone and topiramate, and less consistent benefits have been observed for antidepressants bupropion and mirtazapine.^3^ SUDs and mental disorders have high comorbidity, and the combination of SUD and ADHD is associated with an increase in risk of other psychiatric comorbidities, such as mood, anxiety and personality disorders.^30^ In this study, lisdexamphetamine was associated with beneficial outcomes. Also, the combination of ADHD medications showed a trend toward positive outcomes, although the results were not statistically significant. The use of methylphenidate was associated with the lowest observed mortality. Lisdexamphetamine is licensed for doses ranging from 30 to 70 mg/d in the treatment of ADHD and binge eating disorder in non–stimulant-dependent populations, although there is available safety data from the use of lisdexamphetamine up to 250 mg/d.^24^ In this study, 1511 persons (10.8%) were taking lisdexamphetamine. The most beneficial outcome was observed with doses from 45 to 85 mg/d. Overall, 1657 individuals (11.9%) were diagnosed with ADHD at baseline (n = 3160; 22.6% at the end of study), and the use of lisdexamphetamine might have been indicated for its treatment. However, the use of lisdexamphetamine was associated with positive outcomes in between-analyses also, indicating that it may have potential for improving outcomes in individuals who use methamphetamine in general. Concerning the positive results in treating MAUD with stimulant analogs, it may signalize the possibility to treat MAUD parallel to opioid and tobacco use disorders, in which treatment with agonistlike medication has been successfully implemented.^22^ Naltrexone has been a promising candidate in treating amphetamine use disorder,^3,12,20^ and therefore we analyzed various pharmacological treatments of different SUDs. However, naltrexone had no association with the outcomes of interest in our study. To exclude the impact of possible poor adherence to continue oral naltrexone soon after it is started, we performed a sensitivity analysis for the main outcomes by omitting the first 30 first days of medication use. Still, the use of naltrexone was not associated with a lower risk of hospitalizations or death. It should be noted that this concerned only oral naltrexone, as extended-release injectable naltrexone was not available during the study period. However, the combination of different SUD medications was associated with a lower risk of hospitalization due to SUD and of any hospitalization or death. The finding may be explained by the fact that people with SUDs tend to have comorbidities to other SUDs, and treating different disorders with different medications may lead to better outcomes. The use of buprenorphine was associated with a significantly lower risk of any hospitalization or death and showed a positive trend in reducing SUD hospitalization and all-cause mortality, although the associations were not statistically significant. This result is in line with a recent finding where the use of buprenorphine was associated with a reduction in hospitalizations due to opioid use disorder and all-cause mortality.^31^ Methadone, also used in the treatment of opioid use disorder, was not clearly associated with beneficial outcomes. This may be due to the fact that methadone is associated with more severe adverse effects and a greater risk for sublethal intoxication, which buprenorphine does not have due to its ceiling effect. However, when the outcome was death due to overdose, both buprenorphine and methadone were associated with a lower risk. The mood stabilizer topiramate has been suggested to be beneficial in treating MAUD.^21,32^ In our study, the use of any of the studied mood stabilizers were not associated with a decrease or increase in risk of studied outcomes. In addition, the use of specific antidepressants was not associated with lower risk of hospitalizations or death, which is in line with previous studies,^3,6^ and only the use of mirtazapine in combination with counseling and bupropion in combination with with naltrexone have shown previously positive signals in treating MAUD.^12,13^ In fact, in this study, the use of antidepressants as a group was associated with a statistically significant increase in risk of SUD hospitalization and any hospitalization or death, and the use of mirtazapine and bupropion was not associated with any of the outcomes of interest in our study. Overall, the use of benzodiazepines and antipsychotics was associated with an increase in risk of hospitalizations as well as mortality. Poor outcomes associated with use of benzodiazepines in other SUDs have been recently demonstrated.^31,33^ The antipsychotic aripiprazole has been previously studied in the treatment of amphetamine or methamphetamine dependence and has been found not only ineffective in reducing methamphetamine use, but in fact increasing it.^34,35^

The main strengths of this study are large population size of almost 14 000 persons with nationwide coverage of people with diagnosed MAUD. Previous studies concerning the effectiveness of medications for MAUD are mostly randomized clinical trials limited by small sample sizes, low participant retention, and low treatment adherence rates. The median follow-up time in this study was 3.9 years. Overall, the results are generalizable for real-world patients and offer new and useful information on the association of medications widely used in persons with MAUD with long-term health outcomes. We analyzed the main outcomes by using within-individual design where each individual acts as his or her own control. The method eliminates selection bias by accounting for factors remaining constant for an individual. In addition, we used data on actually purchased medications instead of data on prescriptions given to patients. Drug use was modeled with the PRE2DUP method, which is known to estimate drug use-periods with high accuracy.^36^ We analyzed various medications from different medication groups and performed sensitivity analyses for the most consistent findings, which increases the reliability of the results.

### Limitations

Although within-individual analyses eliminate selection bias, they do not eliminate protopathic bias. In other words, pharmacological treatments are often discontinued when clinical state has improved and are started when clinical state deteriorates. Therefore, the results may underestimate the putative beneficial effect with treatments, and this may partly explain the poor results for antidepressants, benzodiazepines, and antipsychotics. To control for this bias, we conducted sensitivity analyses by omitting the first 30 days of use, and the results were in line with main analyses. One of the limitations of this study is that we had no information on possibly reduced amphetamine or methamphetamine consumption or total abstinence. In addition, there was no information on the possible effects of withdrawal symptoms or craving of amphetamine or methamphetamine. Thus, we evaluated the effectiveness of different medications by estimating the risk for unfavorable outcomes (hospitalizations or death), as these outcomes represent significant disadvantages and costs for both the individual and society. Another limitation of this study is that we did not know how many of the studied medications were indicated for some specific comorbidity. For example, we do not know whether lisdexamphetamine was used to treat ADHD or (off-label) MAUD. However, the positive findings with lisdexamphetamine were consistent in all studied outcomes, encouraging the conducting of randomized clinical trials in the future.

## Conclusions

In this Swedish nationwide cohort study, use of lisdexamphetamine was consistently associated with a reduction in risk of death and hospitalization in persons with amphetamine or methamphetamine. Use of antidepressants were associated with an increase in risk of hospitalization due to SUD and any hospitalization or death. Benzodiazepine use was associated with poor outcomes.

## Notes

Supplement. eMethods eTable 1. Covariate definitions for within-individual and between individual analyses eTable 2. Description of the cohort of persons with (meth)amphetamine use disorder (MAUD) (N= 13 965) eTable 3. The risk for the main outcomes, when first 30 days of medication use was omitted from the analysis eTable 4. Events and adjusted hazard ratios (HRs) with nominal p-values for main outcomes related to use vs. no-use of the most common antidepressants. eTable 5. Adjusted Hazard Ratios (HRs) and 95% confidence intervals (CIs) for the risk of hospitalization due to any cause or death during SUD- and ADHD-medication combinations eTable 6. The risk of death due to overdose (ICD-10-codes X40-X49; X60-X69; Y10-Y19) associated with the use of studied medications eReferences
