# Supplementary material for: Association of Pharmacological Treatments and Hospitalization and Death in Individuals With Amphetamine Use Disorders in a Swedish Nationwide Cohort of 13 965 Patients
Source: JAMA Psychiatry. 2022 Nov 16;80(1):31–9. doi: 10.1001/jamapsychiatry.2022.3788 (PMC9669925; doi:10.1001/jamapsychiatry.2022.3788)
Supplement: Supplement. — eMethods eTable 1. Covariate definitions for within-individual and between individual analyses eTable 2. Description of the cohort of persons with (meth)amphetamine use disorder (MAUD) (N= 13 965) eTable 3. The risk for the main outcomes, when first 30 days of medication use was omitted from the analysis eTable 4. Events and adjusted hazard ratios (HRs) with nominal p-values for main outcomes related to use vs. no-use of the most common antidepressants. eTable 5. Adjusted Hazard Ratios (HRs) and 95% confidence intervals (CIs) for the risk of hospitalization due to any cause or death during SUD- and ADHD-medication combinations eTable 6. The risk of death due to overdose (ICD-10-codes X40-X49; X60-X69; Y10-Y19) associated with the use of studied medications eReferences [file jamapsychiatry-e223788-s001.pdf]

Supplemental Online Content

Hartikainen M, Taipale H, Tanskanen A, Mittendorfer-Rutz E, Lähteenvuo M, Tiihonen J. Association of pharmacological treatments and hospitalization and death in individuals with amphetamine use disorders in a Swedish nationwide cohort of 13 965 patients. *JAMA Psychiatry*. Published online November 16, 2022. doi:10.1001/jamapsychiatry.2022.3788

eMethods

eTable 1. Covariate definitions for within-individual and between individual analyses

eTable 2. Description of the cohort of persons with (meth)amphetamine use disorder (MAUD) (N= 13 965)

eTable 3. The risk for the main outcomes, when first 30 days of medication use was omitted from the analysis

eTable 4. Events and adjusted hazard ratios (HRs) with nominal p-values for main outcomes related to use vs. no-use of the most common antidepressants.

eTable 5. Adjusted Hazard Ratios (HRs) and 95% confidence intervals (CIs) for the risk of hospitalization due to any cause or death during SUD- and ADHD-medication combinations

eTable 6. The risk of death due to overdose (ICD-10-codes X40-X49; X60-X69; Y10-Y19) associated with the use of studied medications

eReferences

This supplemental material has been provided by the authors to give readers additional information about their work.

eMethods

Exposure

Exposure to studied medications was categorized as medications for substance use disorders (SUD), medications for attention-deficit hyperactive disorder (ADHD), mood stabilizers, antidepressants, benzodiazepines and related drugs, and antipsychotics. SUD-medications included disulfiram (N07BB01), acamprosate (N07BB03), naltrexone (N07BB04, only oral available during the study period), methadone (N07BC02) and buprenorphine (N07BC01, N07BC51). ADHD medications were defined as N06BA and specifically as amphetamine, dexamphetamine, modafinil, atomoxetine, methylphenidate and lisdexamphetamine. Mood stabilizers included carbamazepine (N03AF01), valproic acid (N03AG01), lamotrigine (N03AX09) and topiramate (N03AX11). These specific drugs were compared with non-use of the drug class. All specific drugs refer to within-class monotherapies and concomitant use of two or more medication from the class was defined as combination use (e.g. concomitant use of disulfiram and naltrexone). In addition, we analysed the risk of main and secondary outcomes associated with the following drug classes: benzodiazepine and related medications (N05BA, N05CD, N05CF), antidepressants (N06A) and antipsychotics (N05A, including lithium). Sensitivity analyses were conducted for specific antidepressants, including ten most common antidepressants: mirtazapine, sertraline, venlafaxine, escitalopram, bupropion, citalopram, fluoxetine, duloxetine, amitriptyline and paroxetine.

PRE2DUP

Medication use was modelled into drug use periods (when drug use started and ended) based on dispensing data with the PRE2DUP-method (From Prescriptions to Drug Use Periods). The method is based on the calculation of sliding averages of daily dose (in DDDs), the purchased amounts of medications and personal medication use patterns. The method utilizes product level parameters which control the joining of purchases, takes into account hospital stays (when medication use is not recorded in the register) and stockpiling of medications when constructing use periods.[1]

Dose analyses

Additional analyses were conducted for lisdexamphetamine to inform most beneficial dose range. Temporal dose was estimated at each dispensing based on PRE2DUP-modelled data. Dose estimate was calculated by using two previous dispensings and categorized into dose categories (defined daily doses per day translated into milligrams per day dose, <45, 45-<65, 65-<85 and ≥85 mg/day, designed around tablet strengths available and allowing some variation from strictly everyday use).

Study design

Exposure to medications is modelled as time-varying exposure comparing different medications to non-use of medications. The main study design was within-individual which means that use periods are compared to non-use periods within the same person. The within-individual model is a stratified Cox regression model in which each individual forms his or her own stratum. Within each stratum, time is reset to zero after each outcome event. This reduces selection bias of different treatments, i.e. that non-users may be different than users of a certain medication. The method is described in more detail in: Paul D Allison: Fixed Effects Regression Models[2].

Only persons having an event and having variation in exposure status (on medication/off medication) over time contribute to the model in within-individual analysis, whereas all individuals contribute to the between-individual model. Traditional between-individual model was utilized for analyses of mortality, and as sensitivity analyses for the main outcomes. Exposure in between-individual analyses was similarly time-varying and the reference was non-use of the medication class. Dependence between repeated observations was corrected with robust sandwich estimator in between-individual analyses.

© 2022 Hartikainen M et al. *JAMA Psychiatry.*

**eTable 1. Covariate definitions for within-individual and between individual analyses.** The Anatomical Therapeutic Chemical (ATC) classification codes for covariate medications and International Classification of Diseases (ICD) version 10 codes for somatic diseases are described in the table.		
**Covariate**	**Definition**	**Register**
**Within-individual models**		
Order of treatments	Order of treatment continuously updated in the models, categorized as no treatment, 1st, 2nd, 3rd, >3rd	PDR
Time since cohort entry = Time since first (meth)amphetamine use disorder (MAUD) diagnosis	In years, categorized as 0-1, 1-2, 2-3 and >3 years, continuously updated in the models	NPR
**Between-individual models additionally adjusted for the following (in addition to above mentioned covariates)**		
**Other medication use**: defined time-dependently during the follow-up (current use vs. no use currently)		
Opioid analgesics	N02A	PDR
Non-opioid analgesics	N02BE01, M01A	PDR
Cardiovascular medications	C01-C10, excl. C04, C05	PDR
Alimentary tract and metabolism medications	A02, A04AA, A05, A07, A10	PDR
Antiepileptic drugs	N03A excl. valproate, carbamazepine, lamotrigine, lithium	PDR
The number of previous hospitalizations due to MAUD	Main diagnoses of F15; categorized as ≤1, 2-3, >3, updated time-dependently during the follow-up	NPR
**Comorbidities**: updated continuously in the model as “no” before the first diagnoses and “yes” thereafter		
Cardiovascular disease	I00-I99	NPR
Diabetes	E10-E14, or antidiabetic use A10	NPR, PDR
Asthma/COPD	J42-44	NPR
Previous cancer	C01-C99	NPR
Renal disease	N10-N19	NPR
Previous suicide attempt	X60-84, Y10-34 Z728, Z915	NPR
Other substance use disorder than MAUD	F10–F14, F16, F19	NPR
Depression	F32–F33	NPR
Anxiety disorder	F41	NPR
ADHD	F90	NPR
**Sociodemographic factors:** measured at cohort entry		
Age	≤35, 36-55, >55	LISA
Sex	Male or female	LISA
Education (years)	Low (< 9), medium (10 - 12), high (>12), missing	LISA
NPR: National Patient Register, PDR: Prescribed Drug Register, LISA: The Longitudinal Integration Database for Health Insurance and Labor Market Studies		

© 2022 Hartikainen M et al. *JAMA Psychiatry.*

eTable 2. Description of the cohort of persons with (meth)amphetamine use disorder (MAUD) (N= 13 965), including all residents aged 16–65 living in Sweden with registered first-time diagnosis of MAUD (=cohort entry) during 2005-2016.

N=13965	% (n)
**Use of specific drugs at any time after cohort entry**	
**SUD-medications**	20.5 (2856)
Disulfiram	8.0 (1115)
Acamprosate	4.1 (579)
Naltrexone	6.3 (873)
Buprenorphine	4.7 (652)
Methadone	2.6 (368)
≥2 SUD-medications	4.2 (592)
**Antidepressants**	54.0 (7543)
Tricyclic	5.0 (700)
SSRI	31.6 (4411)
SNRI	13.1 (1825)
Mirtazapin	19.7 (2752)
Bupropion	6.9 (960)
Other antidepressants	3.6 (505)
≥2 antidepressants	20.1 (2800)
**Mood stabilizers**	12.2 (1706)
Carbamazepine	4.3(605)
Valproic acid	4.0 (562)
Lamotrigine	4.6 (642)
Topiramate	0.8 (117)
≥2 mood stabilizers	0.8 (114)
**ADHD-medications**	28.2 (3941)
Amphetamine	0.1 (19)
Dexamphetamine	1.9 (268)
Methylphenidate	21.8 (3043)
Modafinil	0.4 (62)
Atomoxetine	6.3 (881)
Lisdexamphetamine	10.8 (1511)
≥2 ADHD-medications	8.5 (1190)
**Benzodiazepines**	43.7 (6101)
**Antipsychotics**	36.3 (5067)

© 2022 Hartikainen M et al. *JAMA Psychiatry.*

Description of the cohort	
**Mean age** (SD)	34.4 (13.0)
**Male gender**	69.3 (9671)
**Year of cohort entry**	
2006	5.6 (785)
2007	5.3 (743)
2008	5.2 (727)
2009	5.2 (731)
2010	4.7 (655)
2011	4.4 (618)
2012	11.9 (1662)
2013	10.3 (1437)
2014	9.6 (1344)
2015	10.3 (1441)
2016	9.4 (1312)
2017	8.6 (1196)
2018	9.4 (1315)
**Country of birth**	
Sweden	87.0 (12145)
Other Europe	6.2 (869)
Rest of the world	6.8 (952)
**Education**	
Low	45.6 (6365)
Medium	42.6 (5951)
High	6.8 (947)
Missing	5.0 (703)
**Family situation**	
Married without children	2.7 (373)
Married with children	5.9 (829)
Single without children	74.2 (10367)
Single with children	3.9 (543)
<20 years living at home	11.1 (1543)
Missing information	2.2 (311)
**Any income from work (year before cohort entry)**	29.1 (4059)
**Unemployment during previous year**	
1 – 180 days	23.6 (3292)
>180 days	6.4 (890)
No unemployment	70.1 (9784)
**On disability pension at cohort entry**	14.9 (2082)
**Sickness absence during a year before cohort entry**	
1-90 days	9.0 (1258)
>90 days	6.4 (889)
No sickness absence	84.6 (11819)

© 2022 Hartikainen M et al. *JAMA Psychiatry.*

**Comorbidities at baseline (ICD-10-code)**	
Alcohol use disorder (F10)	29.2 (4075)
Opioid use disorder (F11)	11.6 (1623)
Cannabis use disorder (F12)	10.8 (1502)
Sedative use disorder (F13)	12.8 (1791)
Cocaine use disorder (F14)	2.6 (360)
Hallucinogen use disorder (F16)	2.1 (299)
Other psychoactive substance use disorder (F19)	33.9 (4728)
Depression (F32–33)	13.2 (1843)
Anxiety disorder (F41)	19.3 (2690)
ADHD (F90)	11.9 (1657)
Previous suicide attempt (X60–84, Y10–Y34, Z728, Z915)	21.1 (2953)
Cardiovascular disease (I00 – I99)	11.0 (1541)
Previous cancer (C01–99)	1.0 (142)
Diabetes (E10–14, or antidiabetic use, ATC-code A10)	2.6 (362)
Asthma/COPD (J42–44)	4.0 (553)

© 2022 Hartikainen M et al. *JAMA Psychiatry.*

eTable 3. The risk for the main outcomes, when first 30 days of medication use was omitted from the analysis. Bolded results are associated with reduced risk of outcome and results written in red color, indicate the increased risk of the outcome studied.

	SUD HOSP (30 days omitted)		ANY HOSP OR DEATH (30 days omitted)	
**Medication(s)**	**Events**	**HR (95%CI)**	**Events**	**HR (95%CI)**
**SUD-medications**				
Disulfiram	313	1.04 (0.90–1.21)	455	1.07 (0.94–1.21)
Acamprosate	181	0.94 (0.78–1.13)	245	0.92 (0.78–1.09)
Naltrexone	178	1.19 (0.98–1.44)	247	1.14 (0.97–1.35)
Buprenorphine	949	1.00 (0.91–1.11)	1132	0.96 (0.88–1.06)
Methadone	1401	1.16 (1.04–1.28)	1669	1.16 (1.05–1.28)
POLY	151	0.86 (0.70–1.06)	180	0.86 (0.71–1.04)
**Antidepressants**	7864	1.09 (1.04–1.14)	13150	1.10 (1.07–1.14)
**Mood stabilizers**				
Carbamazepine	707	0.91 (0.82–1.02)	787	0.96 (0.88–1.05)
Valproic acid	505	**0.87 (0.76–0.99)**	560	1.00 (0.91–1.11)
Lamotrigine	349	0.87 (0.74–1.03)	389	0.97 (0.87–1.09)
Topiramate	63	0.93 (0.66–1.32)	76	1.01 (0.80–1.28)
POLY	48	1.04 (0.70–1.53)	59	0.99 (0.76–1.29)
**ADHD-medication**				
Amphetamine	9	1.18 (0.30–4.58)	24	0.96 (0.46–2.03)
Dexamphetamine	97	0.91 (0.67–1.23)	211	0.96 (0.78–1.19)
Methylphenidate	2295	0.97 (0.91–1.04)	3908	1.01 (0.96–1.06)
Modafinil	17	0.53 (0.27–1.04)	47	0.76 (0.51–1.13)
Atomoxetine	224	0.88 (0.74–1.05)	343	1.00 (0.87–1.15)
Lisdexamphetamine	443	**0.84 (0.73–0.96)**	855	**0.86 (0.78–0.95)**
POLY	203	0.83 (0.68–1.00)	396	**0.86 (0.75–0.99)**
**Benzodiazepines**	6960	1.21 (1.16–1.26)	12337	1.24 (1.20–1.28)
**Antipsychotics**	6183	1.11 (1.06–1.16)	9478	1.13 (1.09–1.17)

© 2022 Hartikainen M et al. *JAMA Psychiatry.*

eTable 4. Events and adjusted hazard ratios (HRs) with nominal p-values for main outcomes related to use vs. no-use of the most common antidepressants.

	**SUD-hospitalization**			**Any hospitalization or death**		
**Medication**	**Users (events)**	**HR (95%CI)**	**p-value**	**Users (events)**	**HR (95%CI)**	**p-value**
Mirtazapine	2766 (1941)	1.08 (1.00–1.15)	0.04	2752 (2766)	1.09 (1.02–1.15)	<0.001
Sertraline	2202 (1194)	1.03 (0.94–1.12)	0.58	2192 (1996)	1.07 (1.00–1.15)	0.06
Venlafaxine	1325 (990)	1.13 (1.02–1.25)	0.02	1322 (1565)	1.17 (1.07–1.26)	<0.001
Escitalopram	1162 (576)	0.91 (0.80–1.03)	0.15	1155 (997)	1.01 (0.92–1.11)	0.85
Bupropion	972 (317)	1.01 (0.86–1.18)	0.85	960 (501)	1.06 (0.94–1.20)	0.35
Citalopram	953 (581)	1.14 (1.00–1.29)	0.05	951 (1070)	1.15 (1.05–1.27)	0.004
Fluoxetine	880 (526)	1.13 (0.99–1.29)	0.07	876 (962)	1.13 (1.02–1.24)	0.02
Duloxetine	656 (426)	1.13 (0.97–1.31)	0.11	649 (700)	1.09 (0.97–1.22)	0.15
Amitriptyline	531 (249)	1.11 (0.93–1.32)	0.26	526 (427)	1.12 (0.98–1.28)	0.11
Paroxetine	270 (237)	1.14 (0.92–1.42)	0.24	270 (355)	1.19 (1.00–1.43)	0.05

eTable 5. Adjusted Hazard Ratios (HRs) and 95% confidence intervals (CIs) for the risk of hospitalization due to any cause or death during SUD- and ADHD-medication combinations.

	**ANY HOSPITALIZATION OR DEATH**	
**Medication combination**	**Events**	**HR (95%CI)**
**Combination, SUD**		
Disulfiram and naltrexone	32	0.92 (0.61–1.37)
Disulfiram and acamprosate	71	0.76 (0.55–1.05)
Disulfiram and buprenorphine	4	0.37 (0.11–1.26)
Disulfiram and methadone	20	0.80 (0.44–1.45)
Acamprosate and naltrexone	41	0.74 (0.51–1.06)
Acamprosate and buprenorphine	4	0.35 (0.10–1.18)
Acamprosate and methadone	12	0.74 (0.37–1.46)
Buprenorphine and methadone	18	0.98 (0.59–1.63)
Other combination	16	0.79 (0.42–1.48)
**Combination, ADHD**		
Dexamphetamine and methylphenidate	46	1.14 (0.71–1.82)
Dexamphetamine and lisdexamphetamine	82	0.74 (0.54–1.00)
Methylphenidate and atomoxetine	107	0.85 (0.67–1.09)
Methylphenidate and lisdexamphetamine	135	0.86 (0.68–1.08)
Atomoxetine and lisdexamphetamine	16	0.99 (0.52–1.87)
Other combination	42	0.95 (0.62–1.45)

© 2022 Hartikainen M et al. *JAMA Psychiatry.*

eTable 6. The risk of death due to overdose (ICD-10-codes X40-X49; X60-X69; Y10-Y19) associated with the use of studied medications. Black bolded results indicate the reduced risk of outcome (p-value <0.05), red bolded indicate the increased risk of studied outcome (p-value <0.05).

	**OVERDOSE DEATH Between-individual model**			
**Medication(s)**	**Events**	**Users**	**Person years**	**HR (95%CI)**
**SUD-medications**				
Disulfiram	7	1125	497	0.87 (0.40–1.89)
Acamprosate	3	585	250	0.77 (0.24–2.50)
Naltrexone	2	890	285	0.50 (0.12–2.06)
Buprenorphine	6	673	1163	**0.32 (0.14–0.73)**
Methadone	8	379	974	**0.44 (0.21–0.93)**
POLY	1	615	199	0.24 (0.03–1.78)
**Antidepressants**	151	7573	13 410	1.24 (1.00–1.54)
**Mood stabilizers**				
Carbamazepine	8	617	698	0.77 (0.38–1.57)
Valproic acid	10	572	566	1.22 (0.64–2.33)
Lamotrigine	9	656	661	1.40 (0.71–2.78)
Topiramate	1	120	117	0.80 (0.11–6.15)
POLY	0	119	53	-
**ADHD-medication**				
Amphetamine	0	19	48	-
Dexamphetamine	0	271	366	-
Methylphenidate	33	3067	5336	**0.60 (0.42–0.85)**
Modafinil	1	62	49	1.66 (0.22–12.7)
Atomoxetine	4	895	383	0.90 (0.34–2.39)
Lisdexamphetamine	5	1540	1446	**0.34 (0.14–0.82)**
POLY	1	1211	606	0.16 (0.02–1.14)
**Benzodiazepines**	155	6132	10 561	**1.74 (1.40–2.17)**
**Antipsychotics**	111	5102	7029	**1.29 (1.02–1.64)**

© 2022 Hartikainen M et al. *JAMA Psychiatry.*

eReferences

1. Tanskanen A, Taipale H, Koponen M, et al. From prescription drug purchases to drug use periods -- a second generation method (PRE2DUP). *BMC Med Inform Decis Mak*. 2015;15(1):21. doi:10.1186/s12911-015-0140-z

2. Allison PD. *Fixed Effects Regression Models.* SAGE; 2009.
